# An Unsupervised Image Stitching Framework via Joint Iterative Optimization of Deformation Estimation, Feature Registration, and Seamless Blending

**DOI:** 10.3390/s26092782

**Published:** 2026-04-29

**Authors:** Baian Ning, Junjie Liu, Haoxin Yu, Qun Lou, Fang Lin, Shanggang Lin

**Affiliations:** College of Artificial Intelligence & Low-Altitude Technology, South China Agricultural University, Guangzhou 510642, China; 20232147004@stu.scau.edu.cn (B.N.); ljj1029@stu.scau.edu.cn (J.L.); 202334410225@stu.scau.edu.cn (H.Y.); qlou@scau.edu.cn (Q.L.)

**Keywords:** image stitching, unsupervised distortion correction, joint optimization, feature matching, seam optimization

## Abstract

Image stitching is a computational technique designed to align and seamlessly fuse multiple overlapping images into a single panoramic image with an extended field of view. It plays a critical role in diverse domains, including mobile photography, autonomous navigation, and visual perception systems. However, most conventional image stitching pipelines implicitly assume that the input images have been pre-corrected for geometric distortions, particularly radial distortion inherent to wide-angle and fisheye lenses. This assumption often fails in practice, as many consumer-grade cameras lack built-in correction or calibration support. Consequently, applying standard image stitching methods to the uncorrected imagery frequently degrades feature correspondence reliability and introduces visible geometric misalignments and seam discontinuities in the final panorama. To overcome these limitations, this paper introduces a task-driven joint iterative optimization framework for image stitching that unifies unsupervised radial distortion correction, distortion-aware feature registration, and seam-aware blending within a single cohesive optimization objective. Specifically, lens distortion parameters are explicitly modeled as learnable variables and embedded into both the geometric registration and seam optimization sub-problems. An efficient closed-loop optimization strategy is then employed to jointly refine distortion parameters, homography estimates, and optimal seam paths in an alternating, mutually reinforcing manner. Implementation-wise, we first propose a calibration-free initial radial distortion estimation method which leverages intrinsic image gradients and epipolar consistency to provide physically plausible initialization for subsequent optimization. During iteration, distortion parameters are progressively refined by integrating robust geometric constraints derived from current feature matches (via RANSAC-based consensus filtering) with photometric consistency cues. Extensive experiments on multiple public benchmarks featuring pronounced radial distortion demonstrate that our method achieves superior stitching fidelity using metrics including PSNR and SSIM. It also confirms enhanced feature matching stability, which outperforms both distortion-agnostic approaches and two-stage pipelines that decouple distortion correction from registration. Furthermore, comprehensive ablation studies quantitatively and qualitatively validate the functional necessity and synergistic contribution of each core module, confirming the design rationale and effectiveness of the proposed joint optimization architecture.

## 1. Introduction

Image stitching, a fundamental topic in computer vision and image processing, aims to seamlessly integrate multiple images with overlapping fields of view into a single wide-angle, high-resolution panoramic image. As a foundational problem in computer vision, image stitching has found broad applications in panoramic imaging, virtual and augmented reality systems, unmanned aerial vehicle (UAV)-based aerial surveying, and intelligent video surveillance [1,2].

In recent years, the widespread adoption and miniaturization of consumer-grade imaging devices, particularly smartphones and action cameras, have led to a substantial increase in image data originating from such platforms. To maximize scene coverage, these devices commonly employ wide-angle lenses. However, wide-angle optics inherently introduce pronounced radial lens distortion, which causes straight-world structures, especially near image boundaries, to appear curved, thereby compromising geometric fidelity and spatial consistency across the image plane [3].

This lens-induced geometric distortion alters both the global spatial distribution and local geometric relationships of image features. Consequently, it degrades the discriminability and repeatability of feature descriptors and impairs correspondence matching reliability, which ultimately undermines the accuracy of inter-image geometric transformation estimation commonly modeled via homography or projective mapping. As a result, stitching artifacts, including structural misalignment, ghosting, and visible seam discontinuities, are frequently observed in the final composite [4].

However, most conventional image stitching pipelines implicitly assume that the input images have been pre-corrected for geometric distortions [5,6,7,8,9]. This assumption often fails in practice, leading to two typical failure modes. The first is global registration failure, which is caused by radial distortion warping feature descriptors, resulting in inaccurate homography estimation and manifesting as structural misalignment throughout the entire panorama. The second is local seam inconsistency, where residual misalignments persist even after rough correction, causing the optimal seam path to traverse areas with significant parallax or color discontinuities after image warping, thereby producing visible blending artifacts.

Against this backdrop, developing robust methods capable of jointly modeling, estimating, and compensating for lens distortion without relying on precise or complete camera calibration has become a critical research frontier. These methods aim to integrate distortion-aware geometric modeling into the core stitching pipeline, achieving synergistic optimization between distortion correction and image alignment.

It is important to note that simply improving either component independently, for instance, adopting a more robust feature descriptor for global registration or applying a more sophisticated seam-cutting algorithm for local alignment, cannot resolve the fundamental issue. The reason is that the distortion parameter k affects both the feature matching stage, by warping descriptors and degrading correspondence quality, and the seam optimization stage, by altering the geometric alignment that defines the difference map. Hence, decoupling distortion correction from the stitching objective inevitably leads to suboptimal results. A joint optimization framework, in which *k*, the homography *H*, and the seam *S* are iteratively refined in a mutually reinforcing manner, is, therefore, necessary to achieve global geometric consistency and local visual seamlessness simultaneously.

It is noteworthy that the idea of jointly optimizing lens distortion parameters with multi-view geometry has been extensively studied in the field of computer vision [10,11]. These pioneering works primarily focused on solving general camera self-calibration or multi-view 3D reconstruction problems. Subsequent work, such as that by Ju et al. [4], further applied this concept to image alignment, allowing for the estimation of distinct distortion parameters for each image. However, these methods essentially remain within the realm of two-view geometric correction and have not been situated within a complete image stitching system whose ultimate goal is to generate visually seamless panoramas that incorporates seam finding and optimization. In the stitching task, optimal geometric alignment does not automatically guarantee the best visual blending quality; the placement of seams and the quality of fusion are crucial.

It should be clarified that the application scenario of this study primarily addresses the following practical boundary conditions: even on devices with calibration data, the unsupervised joint optimization framework proposed here can further enhance stitching quality under conditions of slight calibration parameter drift or imperfect initial correction. Therefore, this research does not negate the existence of built-in correction but rather provides a robust solution, independent of prior calibration information, for the aforementioned broader and more stringent practical conditions.

Furthermore, high-quality image stitching is not only about generating visually pleasing panoramas but also serves as a critical prerequisite for the reliable operation of many advanced visual perception systems. For instance, in autonomous driving or unmanned mining scenarios, obtaining a surround-view panorama through multi-camera stitching is a key input for large-scale scene understanding, object detection, and tracking. However, under nighttime, tunnel, or low-light conditions, where imaging quality is already severely degraded, if the front-end stitched images further suffer from geometric misalignment or seam artifacts caused by uncorrected distortion, the performance of downstream detection networks will be drastically compromised [12]. Therefore, developing a method capable of producing high-quality, geometrically consistent stitching results even under unknown distortion conditions is of paramount importance for ensuring the robustness of the entire visual system in complex environments.

## 2. Related Work

Regarding the issue of image stitching, extensive studies have been conducted in areas such as image registration, deformation model design, and fusion strategies, where significant progress has been achieved.

In the aspects of image registration and deformation modeling, early image stitching methods mostly relied on global projective models for image alignment. However, such models were difficult when balancing the overall consistency and local alignment accuracy when disparities or complex scene structures exist [2,8]. To overcome this problem, researchers proposed various non-rigid stitching methods that preserve content or are locally adaptive. These methods introduce spatially varying transformation models to improve the effect of local geometric alignment.

Among them, the As-Projective-As-Possible (APAP) method achieved effective modeling of local geometric deformations by introducing a local weighting mechanism based on the global projection model [5]. Subsequently, the Shape-Preserving Half-Projective (SPHP) method further improved the local alignment quality while maintaining the overall shape of the image [6]. Building on this, some studies further proposed the adaptive content-preserving stitching methods from the perspective of visual naturalness to reduce visual distortion caused by non-rigid deformations [7].

For the common problems of disparities and dynamic objects in complex scenes, some studies developed seam search and local optimization mechanisms. By alternately optimizing between registration and seam estimation, these methods alleviate the stitching artifacts caused by geometric inconsistency [8]. Such seam-driven or iterative stitching methods demonstrate strong robustness in handling non-ideal image sequences; thus, they have become one of the most important research directions in image stitching in recent years [9,13].

On the other hand, to address the problem of lens distortion, existing research has proposed various distortion correction methods based on camera calibration. These methods achieve high-precision geometric correction by accurately estimating the camera’s distortion parameters using prior information such as calibration plates [14]. Although these methods showed satisfactory performance under controlled conditions, their practical applications are often restricted by the dependency on calibration conditions.

To improve flexibility, some studies began to explore strategies for distortion estimation and correction without calibration. For example, methods based on the geometric prior line straightness, which estimate radial distortion parameters by analyzing the curvature of linear structures in the image, have been widely studied [15,16]. Additionally, with the development of deep learning, some scholars have proposed using unsupervised or self-supervised neural networks to learn the distortion correction mapping, achieving promising performance in specific scenarios [17,18].

In recent years, deep learning has introduced new approaches to image stitching. One line of work focuses on building end-to-end unsupervised deep homography networks, which directly regress homography matrix parameters from image pairs through differentiable image warping and photometric consistency losses, bypassing explicit feature extraction and matching. Another more cutting-edge research direction explores the use of implicit neural representations to model entire scenes. By optimizing a multi-layer perceptron to implicitly represent color and density fields, these methods can simultaneously optimize camera poses, lens distortion, and scene geometry, achieving high-quality novel view synthesis and stitching. These approaches demonstrate strong potential for joint optimization.

However, these deep learning methods typically require large amounts of training data; their generalization ability is constrained by the training data distribution, and their models often lack interpretability. In contrast, the traditional optimization-based joint framework proposed in this paper offers the following unique advantages. First, it requires no training and is applicable to any new scene or new device. Second, its optimization objective is explicit and interpretable, with each term in the energy function having a clear geometric or visual meaning. Third, its modular design facilitates the integration of new distortion models or seam criteria. Therefore, in application scenarios characterized by data scarcity, high demands for interpretability, or the need for rapid adaptation to new imaging modules, the classical optimization framework presented here remains irreplaceable.

Jointly estimating lens distortion parameters and the geometric transformation between images constitutes another closely related research direction. This type of method avoids the error accumulation that may arise from correcting distortion before estimating geometric transformation, often achieving higher consistency. Early works, such as Sawhney [19] and Fitzgibbon [10], systematically incorporated radial distortion parameters as additional variables to be solved alongside the fundamental matrix within an iterative optimization framework, aiming to recover multi-view geometry without relying on calibration objects. The work of McLauchlan [20] further integrated distortion models into bundle adjustment. Leveraging recent advances in polynomial solvers, Byröd et al. [11] proposed more efficient and stable joint estimation methods. The core objective of these methods is to achieve accurate general-purpose camera self-calibration or 3D scene reconstruction.

Closer to the image stitching task is the work by Ju et al. [4], which extended this idea by allowing the estimation of distinct distortion parameters for each image to be stitched and combining this with homography estimation, significantly improving the alignment accuracy between two images. This provides direct inspiration for stitching images with unknown or varying distortions.

However, this paper points out that all the aforementioned works focus on improving the accuracy of two-view geometric alignment. The objective functions they optimize are purely geometric consistency measures. In the complete image stitching task, the final output is a single panorama whose quality depends not only on geometric alignment but also, to a large extent, on seam placement and pixel fusion quality. The point of minimal geometric reprojection error does not necessarily correspond to the visually most seamless seam location. Therefore, limiting joint optimization to distortion parameters and homography matrices while neglecting interaction with the seam optimization process may lead to suboptimal overall stitching results. Constructing a framework that synergistically optimizes all three, enabling distortion correction to directly serve the ultimate task goal of visual seamlessness, remains an open problem.

In summary, despite significant progress in image stitching, distortion correction, and their preliminary joint treatment, existing methods generally suffer from a fundamental limitation: they treat distortion correction and image stitching as sequential or partially coupled independent stages. Self-calibration-based joint optimization methods [4] optimize geometric objectives rather than stitching visual objectives, while mainstream stitching algorithms [5,6,7,8,9,13,21,22] typically assume pre-corrected input images and lack the capability for explicit modeling and online optimization of distortion parameters. This decoupled paradigm has inherent drawbacks. The optimization target of the preprocessing distortion correction stage is not fully aligned with the final stitching stage’s goal (visual seamlessness). Furthermore, it cannot leverage the abundant matching and alignment information generated during the stitching process for feedback-driven optimization of distortion parameters.

Therefore, this paper aims to address this core issue. We propose a task-driven joint iterative optimization framework. Its fundamental innovation lies not in introducing a new distortion model but in redesigning the role of distortion parameters within the stitching system, transforming them into optimizable variables that can be driven in reverse by the stitching task objective. These parameters form a tightly coupled, bidirectional feedback closed-loop system together with homography estimation and seam finding. This framework achieves a shift from geometry-driven to task-driven optimization.

The initial distortion estimator proposed in this paper operates within the same paradigm as these calibration-free geometric methods. Specifically, we adopt the classic pipeline of line detection (via Hough transform), curvature analysis, and robust aggregation, which have been widely validated in the literature [3,15,16,23]. By explicitly situating our estimator within this established tradition, we demonstrate that calibration-free geometric reasoning provides a credible and physically meaningful foundation for the subsequent joint optimization. Unlike prior work that uses distortion estimation only as a preprocessing step, our framework further refines the distortion parameters in a task-driven manner, guided by the stitching objective itself.

In this paper, we address the issue of insufficient robustness of image stitching under unknown lens distortion conditions. By re-examining the image stitching process from an optimization perspective, we break away from the traditional serial pipeline of distortion preprocessing followed by stitching and construct a joint optimization framework that couples distortion parameters, geometric registration, and seam paths. The innovation of this paper does not lie in proposing a new distortion model but in re-designing the role of distortion parameters in the stitching system, making them optimization variables that can be reverse-driven by the stitching task. The innovation points of this paper are summarized as follows:

(1) A unified modeling framework for distortion parameters participating in the stitching objective function is proposed.

Unlike existing methods, which treat distortion correction as an independent preprocessing step, this paper incorporates the radial distortion parameter *k*, the homography matrix *H*, and the seam path *S* into a unified joint objective function to construct a three-variable coupled optimization model *E*(*k*, *H*, *S*).

In this framework, the distortion parameters no longer merely serve the geometric prior of line straightness preservation but directly take the visual consistency of stitching as the optimization objective, achieving a transition from geometric-driven to task-driven optimization.

This modeling approach breaks the staged assumption of the traditional stitching process at the structural level, making distortion estimation an intrinsic variable of the stitching system.

(2) A bidirectional feedback closed-loop mechanism among distortion, registration, and seams is constructed.

This paper establishes an iterative closed-loop optimization structure, forming a bidirectional feedback relationship among the three sub-modules: more accurate distortion correction can improve the stability of feature matching, more stable matching results can improve the estimation of geometric transformation, and more precise registration and seam estimation can provide task-driven reverse supervision signals for distortion parameters. This closed-loop mechanism differs from the unidirectional information flow of traditional pipelines. Instead, it continuously uses the stitching error to reverse-correct distortion parameters during the optimization process, achieving collaborative convergence among these parameters. From a structural perspective, this represents a weak form of geometric–visual joint bundle optimization, yet it can realize self-supervised updates without requiring calibration information.

(3) An online distortion parameter refinement strategy to achieve task-driven dynamic updates is designed.

During the joint optimization process, an online refinement strategy based on the current matching consistency is designed. By locally linearizing the reprojection error with respect to distortion parameters and combining RANSAC robust estimation, the distortion parameters are updated adaptively in each stitching iteration. The key features of this mechanism are as follows: the optimization signal originates from the stitching process itself, the parameter update is consistent with the stitching objective, and no additional calibration data is required. From the perspective of the optimization process, this is a geometric parameter self-correction mechanism driven by the intrinsic stitching task.

The remainder of this paper is organized as follows: Section 2 reviews the related works. Section 3 details the proposed image stitching framework and modules. Section 4 presents the experimental setup, comparative experiments, and real-world application validation. Section 5 concludes the paper.

## 3. Methodology

### 3.1. Relevant Theoretical Foundations

#### 3.1.1. The Basic Process of Image Stitching

The classic image stitching process consists of three stages: preprocessing (geometric/photometric correction), registration (spatial transformation estimation), and fusion (seam detection and blending). This paper focuses on the joint optimization of geometric distortion correction in the preprocessing stage the subsequent steps.

#### 3.1.2. Lens Distortion Model

We employ the Brown–Conrady model [24] to describe radial distortion. For an ideal coordinate point $p={\left(x,y\right)}^{T}$ on the image, its distorted observation coordinates $p_{d}={\left(x_{d},y_{d}\right)}^{T}$ are:

(1) $$x_{d}=x(1+k_{1}r^{2}+k_{2}r^{4})$$

(2) $$y_{d}=y(1+k_{1}r^{2}+k_{2}r^{4})$$

Here, $r^{2}=x^{2}+y^{2}$ represents the square of the radial distance from the point to the image center. $k_{1}$ and $k_{2}$ are the radial distortion coefficients. Barrel distortion corresponds to negative values, whereas pillbox distortion corresponds to positive values. We mainly estimate $k_{1}$, and the model can be extended to higher orders.

#### 3.1.3. Formal Definition of the Problem

Given a pair of images $I_{A}$ and $I_{B}$ with unknown radial distortion, our goal is to jointly estimate the distortion parameters $k={\left[k_{1},k_{2}\right]}^{T}$, the homography matrix *H* between the images, and the optimal stitching seam *S*, such that the final stitched result is visually seamless. This is equivalent to minimizing the joint objective function defined by the reprojection error and the seam inconsistency.

### 3.2. An Image Stitching Method Integrating Unsupervised Distortion Correction

**Figure 1:**
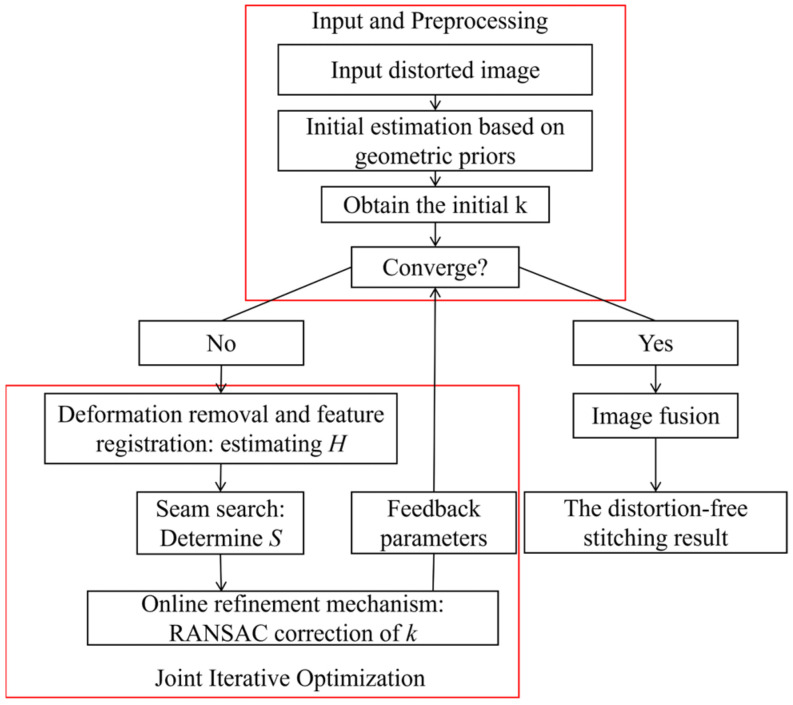
Joint iterative optimization framework for deformation–registration–seam correction.

This section provides a detailed explanation of the core algorithm proposed in this paper. As shown in Figure 1, the proposed method is based on the classic registration-seam iteration framework and introduces a front-end distortion estimation module and an inner-loop distortion parameter refinement mechanism, forming a complete joint optimization pipeline for distortion parameters, registration, and seam. This section describes the design and implementation of each module in detail.

#### 3.2.1. Overall Framework of the Algorithm

The three-branch joint iterative optimization framework proposed in this section is illustrated in Figure 1. Its core idea is to alternately optimize the three sub-modules within the iterative loop and establish a bidirectional feedback mechanism [25]. The key to this framework is the construction of such bidirectional feedback. Accurate distortion correction improves the quality of feature matching, providing a better foundation for registration and seam search; meanwhile, the high-quality matching point pairs and alignment error information generated during registration and seam search provide task-driven self-supervised signals for the refinement of distortion parameters, so that the optimization objective directly serves the final stitching quality.

First, input the original distorted image pairs $I_{A}$ and $I_{B}$. Then, based on the image content, we estimate the initial distortion parameters $k^{\left(0\right)}$ for initialization and correct the images. For the iterative optimization part, in the *t*-th iteration, we use the currently estimated distortion parameters $k^{\left(t\right)}$ to extract and match features on the corrected images and estimate the homography matrix $H^{\left(t\right)}$ via RANSAC. For seam search, we compute the difference map and then obtain the current optimal seam $S^{\left(t\right)}$ through graph-cut. We adopt the classical graph-cut model for seam search, whose energy function consists of color difference terms. We focus on verifying the influence of distortion correction on seam stability, rather than proposing a new seam model. For the distortion refinement part, based on the current matching point pairs $\left\{p_{i},q_{i}\right\}$ and homography matrix $H^{\left(t\right)}$, we optimize and update the distortion parameters to $k^{\left(t+1\right)}$. We then re-correct the original images with $k^{\left(t+1\right)}$ and check whether the objective function converges. If not, we set t=t+1 and continue the iteration. Finally, output the final stitching result ${\;I}_{panorama}$ and the estimated distortion parameters *k*.

#### 3.2.2. Initial Distortion Estimation Based on Image Content

We use the Canny operator and the probabilistic Hough transform [26] to detect line segments. By merging collinear and adjacent short line segments and filtering out those that are too short or have overly scattered directions (which may correspond to natural curves), we obtain a reliable set of straight lines

(3) $$L=\{l_{1},l_{2},\ldots ,l_{i}\}$$

We set the angular tolerance for merging collinear segments to ±3°, and the minimum length threshold for a segment to be retained is 20 pixels. Segments that are shorter than this threshold or have a direction variance exceeding 15° indicate that potential natural curves are discarded. These values were determined empirically and are used consistently across all experiments.

The point set $P_{i}$ on each line $l_{i}$ fits a quadratic curve

(4) $$y={\;a}_{i}{\;x}^{2}+b_{i}\;x+c_{i}$$

where the coefficient $a_{i}$ describes the curvature. According to the distortion model, for an ideal straight line passing near the image center, its curvature *a* is approximately related to the distortion coefficient $k_{1}$ and the average radius ${\bar{r}}_{i}$ of the line by $a_{i}$ ∝ $k_{1\;}$∙${\bar{\;r}}_{i}$. Therefore, we use a robust estimation algorithm. First, for each line $l_{i}$, we construct a nonlinear least squares problem using its point set $P_{i}$ and the distortion model to directly obtain a candidate value of $k_{1}^{\left(i\right)}$. Then, all candidate values {${\;k}_{1}^{\left(i\right)}\;$} from all lines are collected. Finally, outliers are removed via clustering or median filtering, and the cluster center or median is taken as the global initial estimate $k_{1}^{\left(0\right)}$. This method avoids the empirical scaling factor *P* and has a clearer geometric meaning.

It should be noted that the linearized approximation curvature $a_{i}$ ∝ $k_{1\;}$∙${\bar{\;r}}_{i}$ holds accurately only when the radial distortion is mild (|$k_{1\;}$| < 0.1) and the line segment passes near the image center. Under severe barrel distortion ($k_{1}$ < −0.3) or for lines located near image corners, the approximation error increases. However, the purpose of this initial estimation is not to obtain an exact distortion parameter but to provide a physically plausible initialization that prevents the subsequent joint optimization from converging to a poor local minimum. We empirically verified that even under severe distortion, the initial estimate remains within 30% of the ground-truth value for most cases in our datasets, which is sufficient for the alternating optimization to converge to a satisfactory solution.

#### 3.2.3. Joint Iterative Optimization Algorithm

We define the joint objective function as the weighted sum of the feature point reprojection error and the seam smoothness:

(5) $$\;\;\;\;\;\;\;\;\;E(k,H,S)=\sum_{i=1}^{N}{\left||q_{i}\;-\;f\;(p_{i};k,H)|\right|}^{2}\;+\lambda \sum_{(x,y)\in S}D(I_{A}^{w}(x,y;k,H),I_{B}(x,y))$$

Here, $f(p_{i};\;k,H)$ denotes the process of first distorting point $p_{i}$ with parameter *k*, and then projecting it onto the target coordinate system via homography *H*. *D*(∙) is a pixel difference metric, such as color and gradient differences. Since optimizing seam S is a discrete optimization problem, we adopt the following alternating optimization strategy.

The weight parameter λ is used to balance the geometric alignment error and the visual fusion quality. We determined λ = 0.7 through grid search on the validation set, and this value has demonstrated robust performance across multiple datasets.

(1) Fix *k* and *H*; optimize *S*: This is the classic optimal seam-searching problem, solved by the graph-cut algorithm [27].
(2) Fix *S* and *k*; optimize *H*: Select reliable feature points in and around the seam region and minimize the reprojection error using the Levenberg–Marquardt algorithm [28].
(3) Fix *S* and *H*; optimize *k*: This is the key innovative step of this paper. We solve the sub-problem:

(6) $$k^{\left(t+1\right)}=argmin\sum {\left||q_{i}-f\;(p_{i};\;k,H^{\left(t\right)})|\right|}^{2}$$

We adopt a robust estimation algorithm based on RANSAC. First, we perform sampling by randomly selecting four pairs of matching points multiple times and solve for the minimum sample set for $k_{1}$ and $k_{2}$. Then, for a matching point pair $\left(x_{i},y_{i})↔(x_{i}^{\prime },y_{i}^{\prime }\right)$, given the current estimated $k^{\left(t\right)}$ and H, the reprojection error is defined as:

(7) $$r_{i}=y_{i}-f(H\;*\;\phi (x_{i};k))$$

where ϕ denotes the distortion correction function. To solve the distortion parameter increment ∆k, we perform a first-order Taylor expansion of ϕ at $k^{\left(t\right)}$:

(8) $$\phi (x_{i};k^{\left(t\right)}+∆k)\approx \phi (x_{i};k^{\left(t\right)})+J_{i}\;*\;∆k$$

where $J_{i}$ is the Jacobian matrix of ϕ with respect to k, evaluated at $x_{i}$, $k^{\left(t\right)}$. Substituting this into the error function yields a linear least squares problem with respect to ∆k. This problem can be efficiently solved to obtain the candidate parameter $k_{cand\;}$.

We count the number of inliers by computing the symmetric transfer error of all matching points corrected with $k_{cand\;}$. We then select the model with the largest number of inliers and use all inliers to perform nonlinear optimization based on the Gauss–Newton method [29] to obtain the updated $k^{\left(t+1\right)}$ for the current iteration. Next, we re-correct the original image $I_{A}$ using the updated $k^{\left(t+1\right)}$. Feature points must be re-extracted and re-matched on the corrected image to ensure geometric consistency. Although this increases the computational cost, it guarantees the reliability of the optimization foundation. RANSAC is still used within each iteration to resist mismatched outliers caused by imperfect registration and seam estimation, thus ensuring the robustness of the k update.

The alternating optimization strategy we employ belongs to the block coordinate descent method. Although the objective function E(k, H, S) is not smooth everywhere due to the inclusion of the discrete seam search term, when any two variables are fixed, optimizing the third sub-problem ensures that the corresponding sub-objective monotonically decreases or reaches its optimum. Furthermore, both the reprojection error term and the seam difference term have lower bounds. Therefore, the entire iterative process guarantees that the sequence of objective function values is non-increasing and bounded below, enabling rapid convergence to a stable solution in practice (as demonstrated in Section 3.2.4). A rigorous theoretical analysis of convergence to a stationary point requires more complex considerations and is left for future work.

#### 3.2.4. Convergence Analysis

The alternating optimization strategy described in Section 3.2.3 belongs to the class of block coordinate descent methods. While the objective function *E*(*k*, *H*, *S*) is non-convex and includes a discrete seam search term, the following properties hold:

(1) Boundedness: All three terms in Equation (5) are non-negative. The reprojection error term is zero at the global optimum, and the seam smoothness term is bounded below by zero. Therefore, *E*(*k*, *H*, *S*) ≥ 0.
(2) Monotonic decrease: Each sub-step in the alternating optimization, which fixes two variables and solves for the third, ensures that the corresponding sub-objective does not increase. For the sub-steps involving H and k, the Levenberg–Marquardt and Gauss–Newton methods guarantee a non-increasing objective. For the seam sub-step, graph-cut finds the global optimum, thus minimizing the seam cost for fixed *k* and *H*.
(3) Empirical validation: As shown in our experiments, the objective function typically converges within 3 to 5 iterations. The relative decrease drops below 10^−4^ after 4 iterations on average. Thanks to the good initialization provided by our line-based estimator (Section 3.2.2), the optimization rarely encounters poor local minima.

We acknowledge that a rigorous theoretical convergence proof for non-convex block coordinate descent with discrete variables is beyond the scope of this work. However, as demonstrated in similar iterative refinement frameworks [30], the convergence of such schemes depends critically on initialization quality and the smoothness of the residual landscape, both of which are carefully addressed in our design.

### 3.3. Algorithm Pseudocode

The overall procedure of the proposed joint iterative optimization framework, as described in Section 3.2, is summarized in Algorithm 1. The algorithm begins with an unsupervised initial distortion estimation based on image line structures. It then enters an alternating optimization loop, where in each iteration, feature matching and homography estimation are performed on the distortion-corrected images, followed by seam search via graph-cut, and finally online distortion parameter refinement using RANSAC-based robust estimation. The loop terminates when the objective function converges or the maximum number of iterations is reached. The final stitching result is produced by blending the optimally corrected and warped images along the computed seam path.
**Algorithm 1:** Image stitching algorithm incorporating unsupervised distortion correctionInput: Original images $I_{A}$ and $I_{B}$

Output: Stitched result $I_{\mathrm{pano}}$, Distortion parameters k

1: $k^{\left(\theta \right)}$ ← $\mathrm{UnsupervisedInitialEstimation}(I_{A},{\;I}_{B})$//Section 3.1

2: $I_{A}\_\mathrm{corr}$ ← $\mathrm{Undistort}(I_{A},\;k^{\left(\theta \right)})$

3: for *t* = 0 to $T_{\max}$ − 1 do

4:   //1. Registration

5:     $feat1,desc1\leftarrow \mathrm{ExtractFeatures}(I_{A}\_\mathrm{corr})$

6:     $feat2,desc2\leftarrow \mathrm{ExtractFeatures}(I_{B})$

7:     $\left\{p_{i},q_{i}\right\}$, $H^{\left(t\right)}$ ← $\mathrm{FeatureMatchingAndRANSAC}(I_{A}\_\mathrm{corr},\;I_{B})$

8:   //2. Seam search

9:      $I_{A}\_$warp ← $\mathrm{Warp}(I_{A}\_\mathrm{corr},\;H^{\left(t\right)})$

10:      $D\;\leftarrow \;\mathrm{ComputeDifferenceMap}(I_{A}\_\mathrm{warp},\;I_{B})$

11:      $S^{\left(t\right)}\;\leftarrow \;\mathrm{GraphCut}(D)$

12:   //3. Distortion refinement

13:     $k_{\mathrm{candidates}}$ ← []

14:     for j = 1 to M do//RANSAC loop

15:       sample ← RandomSample$\left(\{p_{i},\;q_{i})\right\}$, 4)

16:       $k_{\mathrm{cand}}$ ← SolveDistortionFromSample(sample, $H^{\left(t\right)},\;k^{\left(t\right)}$)

17:       inliers ← EvaluateInliers
$(\{p_{i},q_{i}\},\;k_{\mathrm{cand}},\;H^{\left(t\right)}$)

18:       $k_{\mathrm{candidates}}$. append(($k_{\mathrm{cand}}$, count(inliers)))

19:     end for

20:     $k_{\mathrm{best}}$← SelectBestModel$\left(k_{\mathrm{candidates}}\right)$

21:     $k^{(t+1)}$ ← NonlinearRefinement$(k_{\mathrm{best}}$, all_inliers, $H^{\left(t\right)})$//Gauss-Newton method

22:   //4. Prepare for the next iteration

23:     $I_{A}\_\mathrm{corr}$ ← Undistort($I_{A}$, $k^{(t+1)}$)//Re-correct the original image using the updated parameters

24:     if ConvergenceCheck$\left(E,\;k^{\left(t+1\right)},\;k^{\left(t\right)},\;S^{\left(t\right)}\right)$ then

25:       break

26:     end if

27: end for

28: $I_{\mathrm{pano}}$ ← Blend$\left(I_{A}\_\mathrm{corr},\;I_{B},\;H^{\left(t\right)},\;S^{\left(t\right)}\right)$

29: reture $I_{\mathrm{pano}},\;k^{(t+1)}$


## 4. Experiments

### 4.1. Dataset

UDIS-D is a large-scale image dataset [31] designed for image stitching and image registration tasks. It contains images with various overlap ratios, different disparity levels, and diverse scenes, including indoor, outdoor, night, low-light, snowy, and zoomed-out scenarios.

The Small Unmanned Aerial Vehicle Image Registration Dataset (SUIRD) is a public dataset [32] for image registration and matching research. SUIRD v1.0 contains 50 image pairs with corresponding ground truth. These image pairs exhibit horizontal, vertical, and mixed viewpoint changes, leading to challenges such as low overlap, image distortion, and severe outliers.

### 4.2. Implementation Details

All experiments were conducted on a platform with Ubuntu 20.04 LTS (Canonical Ltd., London, UK) and an NVIDIA GeForce RTX 3090 GPU (NVIDIA Corporation, Santa Clara, CA, USA). The PyTorch 1.12.1 deep learning framework (Meta AI, Menlo Park, CA, USA) was adopted. Input images were resized to 1200 × 800 pixels. The image processing and feature extraction were implemented using OpenCV 4.8.0 (OpenCV team, Menlo Park, CA, USA). Numerical computations were performed with NumPy 1.23.5 (NumPy community, USA) and SciPy 1.9.3 (SciPy community, USA). During training, we focused on comparing our method with state-of-the-art (SOTA) approaches. The maximum training iteration $T_{\max}$ was set to 5 with λ=0.7 and $\epsilon_{E}=10^{-4}$.

### 4.3. Evaluation Metrics

Peak Signal-to-Noise Ratio (PSNR) [33]: A classical objective metric based on pixel error. It calculates the mean squared error (MSE) between the stitched image and the reference images and then computes the logarithm of the ratio between the maximum pixel value and the MSE, with the unit of decibels (dB). A higher PSNR value indicates more accurate pixel-level reconstruction.

Structural Similarity Index (SSIM) [33]: Measures the similarity between the reference and stitched images in local windows, considering luminance, contrast, and structure. The output score ranges from 0 to 1, where a value closer to 1 indicates better structural and perceptual consistency with the ground truth.

Feature matching inlier ratio [34]: Used to evaluate geometric consistency, which can indirectly reflect the improvement of distortion correction on feature matching stability. A higher value yields better performance.

### 4.4. Comparative Experiments

#### 4.4.1. Quantitative Comparison

We compared the proposed method with several SOTA methods, including APAP [5], AANAP [7], and seam-driven [25]. Experiments were conducted on two public datasets: UDIS-D and SUIRD. We evaluated several objective metrics (PSNR, SSIM, and inlier ratio) to verify the effectiveness of the proposed method.

As shown in Table 1, our method achieves the best performance among all compared approaches on all metrics across both datasets. SOTA methods such as APAP and AANAP perform relatively poorly because they cannot effectively handle complex deformations. Our method yields significant improvements over these methods, which strongly demonstrates that the proposed joint iterative optimization is superior to independently optimizing any single component.

#### 4.4.2. Visual Comparison

**Figure 2:**
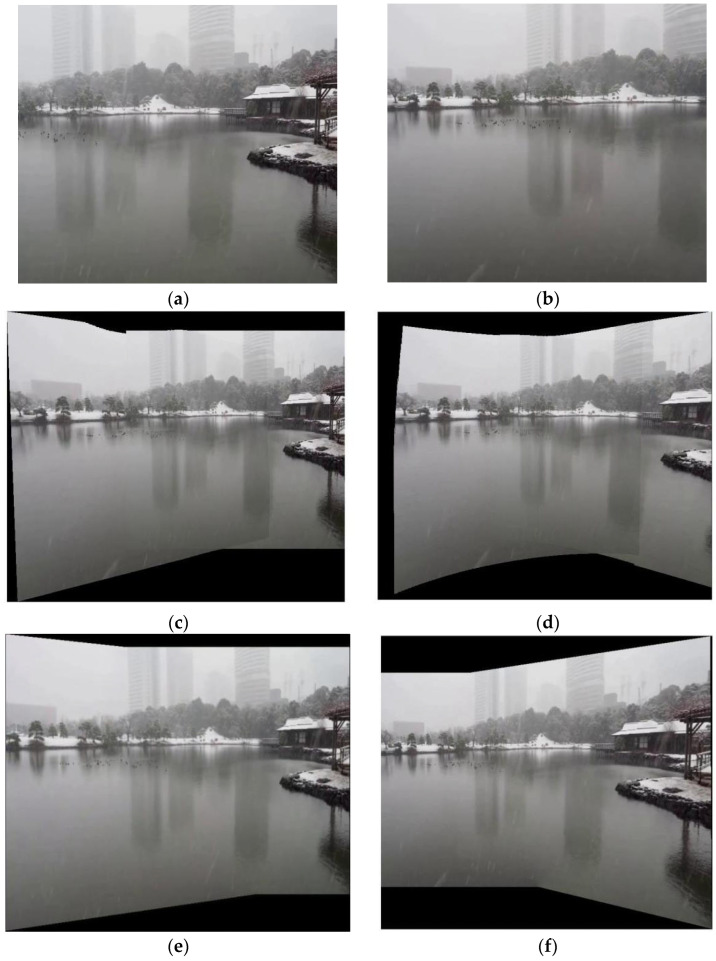
Stitching results of different comparison methods on the UDIS-D dataset. (**a**,**b**) Input image; (**c**) APAP; (**d**) AANAP; (**e**) seam-driven; (**f**) ours.

Quantitative comparisons cannot fully reflect visual perception quality. Therefore, we provide detailed visual comparisons, as depicted in Figure 2.

We can observe the following phenomena. As shown in Figure 2c,d, the stitching results obtained by the APAP and AANAP methods suffer from obvious ghosting and blurring, with severe structural misalignments. The result of the seam-driven method in Figure 2e achieves good global alignment and roughly consistent contours, but local ghosting still exists. In contrast, our result in Figure 2f achieves the best visual stitching performance. It not only preserves global geometric correctness but also obtains nearly perfect alignment in local regions through iterative optimization. The stitching seam avoids all misaligned regions, producing the most visually consistent and natural result.

For efficiency, we computed the average running time of each method. The running times of the APAP, AANAP, and seam-driven methods are 2.1, 3.5, and 4.8 s, respectively, while our method takes 8.7 s per iteration. Our method clearly trades computational efficiency for stitching accuracy. Nevertheless, the improvement in quantitative metrics is significant.

Each iteration of our method takes approximately 8.7 s, with the primary overhead stemming from the re-extraction and matching of features after each update of the distortion parameters. This ensures the absolute reliability of feature correspondences following changes in geometric deformation. However, in practical applications, to balance efficiency and accuracy, the following strategy can be adopted. After the mid-phase of iterations, the feature point locations from the previous iteration can be used as initialization, with only local descriptor lightweight matching performed. Preliminary experiments indicate that this strategy can reduce the time per iteration by approximately 30%, while having minimal impact on the final stitching quality. For scenarios with extremely high real-time requirements, this optimization is crucial, and we will pursue this direction in future work.

#### 4.4.3. Synthetic Distortion Recovery Experiment

To directly measure the accuracy of distortion parameter recovery, we conducted an auxiliary experiment using the UDIS-D dataset. We first undistorted all images using ground-truth distortion parameters that were obtained from existing metadata. We then synthetically applied known radial distortion ($k_{1\;}$ ϵ {−0.3, −0.2, −0.1, 0.1, 0.2, 0.3}) to these undistorted images. Our method was then applied to estimate $k_{1\;}$ from the synthetically distorted image pairs. The recovery error is measured as |$k_{1}$_estimated − $k_{1}$_gt|.

As shown in Table 2, the average recovery error is below 0.02 for mild distortion (|$k_{1\;}$| ≤ 0.2) and below 0.05 for severe distortion (|$k_{1\;}$| = 0.3). This confirms that our joint optimization framework can accurately recover distortion parameters without requiring explicit calibration.

### 4.5. Ablation Studies

To verify the contributions of each component in the proposed framework, we conducted ablation experiments. We constructed three variants:

(i) Ours w/o IE: Without the initial distortion estimation module.
(ii) Ours w/o OR: Distortion correction is only applied initially; the distortion parameter *k* is fixed during the original registration-seam iteration framework, i.e., without online refinement of *k*.
(iii) Ours (Full): The complete proposed framework.

The comparison results, shown in Table 3, demonstrate that the initial distortion estimation module can significantly improve feature matching stability in the early stages of iteration, while the online refinement module mainly contributes to the fine adjustment of geometric consistency in the later stage.

**Figure 3:**
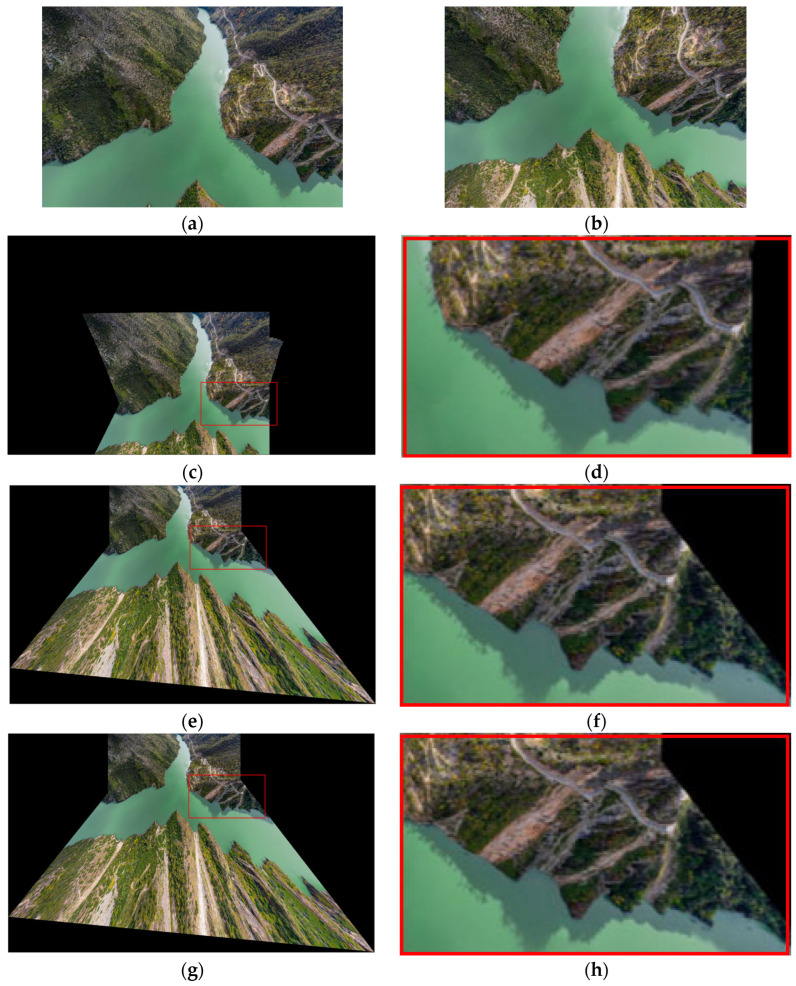
Visual comparison of stitching results for different ablation variants on the SUIRD dataset. (**a**,**b**) Input images; (**c**,**d**) ours w/o IE; (**e**,**f**) ours w/o OR; (**g**,**h**) ours (full).

The qualitative results are illustrated in Figure 3. We show the output differences of each variant using a typical example. As indicated by the red box, ours w/o IE yields obvious ghosting due to the absence of the initial distortion estimation module. Ours w/o OR alleviates ghosting but fails to completely remove it because the distortion parameter *k* is not refined online. Only the full model successfully eliminates all visual artifacts and produces the sharpest and best-aligned results. The qualitative observation is well supported by the quantitative results in Table 2, where the full model achieves the best scores on both datasets.

### 4.6. Sensitivity Analysis of the Weight Parameter λ

We conducted a sensitivity analysis on the key parameter λ in the joint optimization to validate the robustness of its value selection. As shown in Table 4, we tested different values of λ ranging from 0.1 to 1.5 on the UDIS-D validation set. The results indicate that within the range of λ = 0.5 to 0.9, both PSNR and SSIM metrics remain stable, with fluctuations of less than 1%. When *λ* = 0.7, all metrics reach or approach their peak values, and the number of convergence iterations is minimized. This demonstrates that our method is insensitive to variations in *λ*, and *λ* = 0.7 is a reliable optimal choice. Therefore, in all comparative and ablation experiments, we fixed *λ* = 0.7.

## 5. Conclusions

This paper addresses the problem of image stitching under unknown camera distortion and proposes an innovative unsupervised joint iterative optimization framework. The main contributions are as follows: (1) A robust initial distortion estimator based on geometric priors is designed; (2) a unified iterative optimization framework is constructed for distortion parameters, homography matrix, and seam path; and (3) an online distortion refinement mechanism based on RANSAC is implemented, which mutually reinforces distortion correction and image stitching. Experiments demonstrate that the proposed method can achieve stitching quality close to that of an ideal calibration scheme without explicit calibration. The key difference between our method and naive pipeline lies in the bidirectional feedback mechanism: distortion parameters are optimized under the guidance of stitching objective, rather than relying solely on geometric prior constraints.

Although the framework proposed in this paper has achieved promising results in uncalibrated image stitching, it still has certain limitations, which point to directions for future research.

(1) Extension to Multi-Image Stitching: The current framework is designed for image pairs, and the distortion parameter *k* is estimated independently for each pair. Extending it to multi-image panoramic sequences requires ensuring consistency in distortion parameter estimation across different image pairs. A natural extension would be to incorporate a globally consistent distortion parameter *k* into a unified bundle adjustment framework. This would simultaneously minimize the reprojection errors and seam inconsistencies across all overlapping regions while enforcing consistency of the distortion model across all images.
(2) More Comprehensive Lens Model: This paper primarily addresses radial distortion. However, consumer-grade wide-angle lenses often exhibit tangential distortion, vignetting, and chromatic aberration, all of which can affect the visual consistency of stitching boundaries. Future work will explore integrating a more complete lens model and investigate the joint optimization of photometric and geometric corrections.
(3) Scene Dependency of the Initial Estimator: Our initial estimator relies on the presence of sufficient linear structures in the scene. Its performance may degrade in natural texture scenes lacking distinct linear features such as dense foliage, water surfaces, or highly textured indoor scenes. To address this limitation, we propose two fallback strategies for future work. First, when fewer than 20 reliable line segments are detected, we can infer an initial *k* by minimizing the symmetric transfer error across tentative feature matches, assuming that the optimal *k* should minimize the residual of the homography estimation. Second, a lightweight convolutional neural network can be trained to predict *k* directly from image patches. Although this introduces a dependency on training data, it can serve as a reliable fallback when geometric priors are insufficient.

Understanding the scene-dependent variability in structural cue availability, as highlighted in the context of geometric reasoning for natural image analysis [17], is crucial for developing robust initialization strategies. We aim to explore hybrid approaches that combine geometric and learning-based cues in future work.

This work advances the joint optimization of unsupervised distortion correction and image stitching. We believe that this work and future explorations will bring new opportunities and vitality to the fields of computer vision and image processing.

## Figures and Tables

**Table 1:** Experimental results of different methods on the UDIS-D and SUIRD datasets (best results are illustrated in bold).

Method	Dataset	PSNR (dB)	SSIM	Inlier Ratio
APAP	UDIS-D	25.31	0.882	74.6
AANAP	UDIS-D	25.85	0.889	77.1
Seam-driven	UDIS-D	28.92	0.894	78.8
Ours	UDIS-D	**29.17**	**0.902**	**81.4**
APAP	SUIRD	24.88	0.865	68.2
AANAP	SUIRD	25.10	0.871	70.5
Seam-driven	SUIRD	27.45	0.880	75.3
Ours	SUIRD	**28.05**	**0.891**	**79.1**

**Table 2:** Distortion parameter recovery error on synthetic data.

True $k_{1}$	Estimated $k_{1}$ (Mean)	Absolute Error
−0.3	−0.278	0.022
−0.2	−0.191	0.009
−0.1	−0.095	0.005
0.1	0.103	0.003
0.2	0.209	0.009
0.3	0.316	0.016

**Table 3:** Ablation experiments on the UDIS-D and SUIRD datasets (the best results are indicated in bold).

Model	Dataset	PSNR (dB)	SSIM
Ours w/o IE	UDIS-D	26.45	0.889
Ours w/o OR	UDIS-D	27.88	0.912
Ours (Full)	UDIS-D	**27.95**	**0.915**
Ours w/o IE	SUIRD	25.60	0.872
Ours w/o OR	SUIRD	27.20	0.885
Ours (Full)	SUIRD	**27.65**	**0.892**

**Table 4:** Sensitivity analysis experiment on the UDIS-D dataset (the best results are indicated in bold).

λ	PSNR (dB)	SSIM	Iterations
0.1	27.30	0.905	13
0.3	27.52	0.908	11
0.5	27.80	0.911	9
0.7	**27.88**	**0.912**	**8**
0.9	27.85	0.911	9
1.1	27.70	0.909	10
1.3	27.55	0.907	12
1.5	27.40	0.906	13

## Data Availability

The data presented in this study are available in UDIS-D at 10.1109/ICASSP48485.2024.10447800 [31] and SUIRD at 10.3390/rs15133379 [32].

## References

[1] Brown M., Lowe D.G. (2007). Automatic panoramic image stitching using invariant features. Int. J. Comput. Vis..

[2] Szeliski R. (2007). Image alignment and stitching: A tutorial. Found. Trends Comput. Graph. Vis..

[3] Devernay F., Faugeras O. (2001). Straight lines have to be straight. Mach. Vis. Appl..

[4] Ju M.H., Kang H.B. (2014). Stitching images with arbitrary lens distortions. Int. J. Adv. Robot. Syst..

[5] Zaragoza J., Chin T.J., Brown M.S., Suter D. (2013). As-projective-as-possible image stitching with moving DLT. 2013 IEEE Conference on Computer Vision and Pattern Recognition.

[6] Chang C.H., Sato Y., Chuang Y.Y. (2014). Shape-preserving half-projective warps for image stitching. 2014 IEEE Conference on Computer Vision and Pattern Recognition.

[7] Lin C.C., Pankanti S.U., Natesan Ramamurthy K., Aravkin A.Y. (2015). Adaptive as-natural-as-possible image stitching. 2015 IEEE Conference on Computer Vision and Pattern Recognition.

[8] Gao J., Kim S.J., Brown M.S. (2011). Constructing image panoramas using dual-homography warping. CVPR 2011.

[9] Lin K., Jiang N., Cheong L.F., Do M., Lu J. (2016). Seagull: Seam-guided local alignment for parallax-tolerant image stitching. European Conference on Computer Vision.

[10] Fitzgibbon A.W. (2001). Simultaneous linear estimation of multiple view geometry and lens distortion. Proceedings of the 2001 IEEE Computer Society Conference on Computer Vision and Pattern Recognition. CVPR 2001.

[11] Byröd M., Brown M., Åström K. (2009). Minimal solutions for panoramic stitching with radial distortion. The 20th British Machine Vision Conference.

[12] Essien E., Frimpong S. (2025). Enhancing autonomous truck navigation in underground mines: A review of 3D object detection systems, challenges, and future trends. Drones.

[13] Zhang F., Liu F. (2014). Parallax-tolerant image stitching. 2014 IEEE Conference on Computer Vision and Pattern Recognition.

[14] Zhang Z. (2000). A flexible new technique for camera calibration. IEEE Trans. Pattern Anal. Mach. Intell..

[15] Alemán-Flores M., Alvarez L., Gomez L., Santana-Cedrés D. (2014). Automatic lens distortion correction using one-parameter division models. Image Process. Line.

[16] Zhang L., Shang H., Wu F., Wang R., Sun T., Xie J. (2019). Robust line-based radial distortion estimation from a single image. IEEE Access.

[17] Rong J., Huang S., Shang Z., Ying X. (2016). Radial lens distortion correction using convolutional neural networks trained with synthesized images. Asian Conference on Computer Vision.

[18] Han D., Chen L., Guo Z., Yang C. (2022). A fisheye distortion correction method based on deep learning. 2022 6th International Conference on Electronic Information Technology and Computer Engineering.

[19] Sawhney H.S., Kumar R. (2002). True multi-image alignment and its application to mosaicing and lens distortion correction. IEEE Trans. Pattern Anal. Mach. Intell..

[20] McLauchlan P.F., Jaenicke A. (2002). Image mosaicing using sequential bundle adjustment. Image Vis. Comput..

[21] Nie L., Lin C., Liao K., Liu S., Zhao Y. (2021). Unsupervised deep image stitching: Reconstructing stitched features to images. IEEE Trans. Image Process..

[22] Zhao Q., Ma Y., Zhu C., Yao C., Feng B., Dai F. (2021). Image stitching via deep homography estimation. Neurocomputing.

[23] Hittawe M.M., Sidibé D., Mériaudeau F. (2015). A machine vision based approach for timber knots detection. Twelfth International Conference on Quality Control by Artificial Vision 2015.

[24] Lelowicz K. (2019). Camera model for lens with strong distortion in automotive application. 2019 24th International Conference on Methods and Models in Automation and Robotics (MMAR).

[25] Liao T., Chen J., Xu Y. (2019). Quality evaluation-based iterative seam estimation for image stitching. Signal Image Video Process..

[26] Song W., Li P., Wang M. (2024). Transmission line detection based on improved hough transform. arXiv.

[27] Chen X., Pan L. (2018). A survey of graph cuts/graph search based medical image segmentation. IEEE Rev. Biomed. Eng..

[28] Fischer A., Izmailov A.F., Solodov M.V. (2024). The Levenberg–Marquardt method: An overview of modern convergence theories and more. Comput. Optim. Appl..

[29] Wang Y. (2012). Gauss–newton method. Wiley Interdiscip. Rev. Comput. Stat..

[30] Beya O., Hittawe M.M., Alashkar T., Fauvet E., Laligant O. (2015). Applying non linear approach for ecg denoising and waves localization. 2015 11th International Conference on Signal-Image Technology & Internet-Based Systems (SITIS).

[31] Feng C.B., Zhang J., Li J., Zhou Y. (2024). Seam mask guided partial reconstruction with quantum-inspired local aggregation for deep image stitching. ICASSP 2024—2024 IEEE International Conference on Acoustics, Speech and Signal Processing (ICASSP).

[32] Liu J., Liang A., Zhao E., Pang M., Zhang D. (2023). Homography matrix-based local motion consistent matching for remote sensing images. Remote Sens..

[33] Hore A., Ziou D. (2010). Image quality metrics: PSNR vs. SSIM. 2010 20th International Conference on Pattern Recognition.

[34] Falkenhagen U., Kössler W., Lenz H.J. (2019). A likelihood ratio test for inlier detection. Workshop on Stochastic Models, Statistics and Their Application.

